# Strong Coupling of Two-Dimensional Excitons and Plasmonic Photonic Crystals: Microscopic Theory Reveals Triplet Spectra

**DOI:** 10.1021/acsphotonics.3c01208

**Published:** 2024-03-27

**Authors:** Lara Greten, Robert Salzwedel, Tobias Göde, David Greten, Stephanie Reich, Stephen Hughes, Malte Selig, Andreas Knorr

**Affiliations:** † Institut für Theoretische Physik, Technische Universität Berlin, 10623 Berlin, Germany; ‡ Institut für Theoretische Physik, Technische Universität Berlin, 10623 Berlin, Germany; § Experimentelle Festkörperphysik, Freie Universität Berlin, 14195 Berlin, Germany; ∥ Department of Physics, Engineering Physics and Astronomy, Queen’s University, Kingston, Ontario K7L 3N6, Canada

**Keywords:** 2D semiconductors, transition metal dichalcogenides, excitons, plasmonic crystals, strong coupling, light–matter interactions

## Abstract

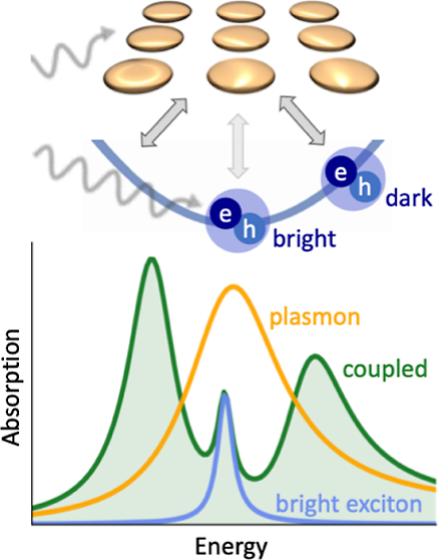

Monolayers of transition metal dichalcogenides (TMDCs) are direct-gap semiconductors with strong light–matter interactions featuring tightly bound excitons, while plasmonic crystals (PCs), consisting of metal nanoparticles that act as meta-atoms, exhibit collective plasmon modes and allow one to tailor electric fields on the nanoscale. Recent experiments show that TMDC-PC hybrids can reach the strong-coupling limit between excitons and plasmons, forming new quasiparticles, so-called plexcitons. To describe this coupling theoretically, we develop a self-consistent Maxwell-Bloch theory for TMDC-PC hybrid structures, which allows us to compute the scattered light in the near- and far-fields explicitly and provide guidance for experimental studies. One of the key findings of the developed theory is the necessity to differentiate between bright and originally momentum-dark excitons. Our calculations reveal a spectral splitting signature of strong coupling of more than 100 meV in gold-MoSe_2_ structures with 30 nm nanoparticles, manifesting in a hybridization of the plasmon mode with momentum-dark excitons into two effective plexcitonic bands. The semianalytical theory allows us to directly infer the characteristic asymmetric line shape of the hybrid spectra in the strong coupling regime from the energy distribution of the momentum-dark excitons. In addition to the hybridized states, we find a remaining excitonic mode with significantly smaller coupling to the plasmonic near-field, emitting directly into the far-field. Thus, hybrid spectra in the strong coupling regime can contain three emission peaks.

## 1 Introduction

The light–matter interaction strength in transition metal dichalcogenide (TMDC) monolayers has been reported to be extremely strong,^1^ e.g., as demonstrated by absorption rates of up to 10% in the visible spectrum.^2,3^ Such a high absorption is particularly noteworthy given the two-dimensional nature of these materials, which possess a thickness of less than 1 nm. In addition to featuring a direct band gap,^2^ TMDC monolayers support in-plane exciton formation due to their two-dimensional structure.^4^ Excitons (bound electron–hole pairs) therefore dominate the optical spectrum below the band edge.^5^ In addition, the remarkably thin nature of the TMDC monolayers results in an increased sensitivity to surrounding materials. Consequently, the atomically thin materials can easily be influenced by various factors, such as the choice of the substrate material, defects,^6,7^ and functionalization,^8^ e.g., with molecules^9,10^ and heterostructure configurations.^11,12^

In contrast, the optical response of metal nanoparticles (MNPs) is dominated by localized plasmons which are collective electron oscillations formed within the metal conduction band.^13^ A special feature of MNPs is a significant amplification of the electric near-field, which additionally allows for manipulating the electric field on dimensions far below the diffraction limit.^14,15^ Arranging MNPs as meta-atoms in a crystal structure yields a plasmonic crystal (PC) with extraordinary strong light–matter interaction. The localized plasmons couple with the electric field and form plasmon–polaritons which can propagate within the crystal^16,17^ and sharpen the single particle plasmon resonance. The optical properties of PCs strongly depend on a variety of parameters, such as lattice structure and nanoparticle shape. By manipulating these, it is possible to tune the optical properties of the crystal over a wide range.^16,18−20^ The strong tunability and enhancement of the electric field make periodic plasmonic structures appealing for light harvesting and nonlinear optics, yielding, e.g., applications for nanoscale lasing^19,21^ and advanced optical spectroscopy.^22,23^

It has also been shown that the absorption of graphene is significantly enhanced by depositing plasmonic nanostructures near the graphene layer.^24,25^ An interaction between localized surface plasmons of a single MNP and excitons in the semiconductor plane can reach the strong coupling regime^26−33^ and absorption rates up to 90%.^34^ This motivates that combining the particular properties of PCs with the environment-sensitive semiconductor monolayer promises a further increase of the light–matter-interaction of the excitons in the TMDC.^35^

**Figure 1:**
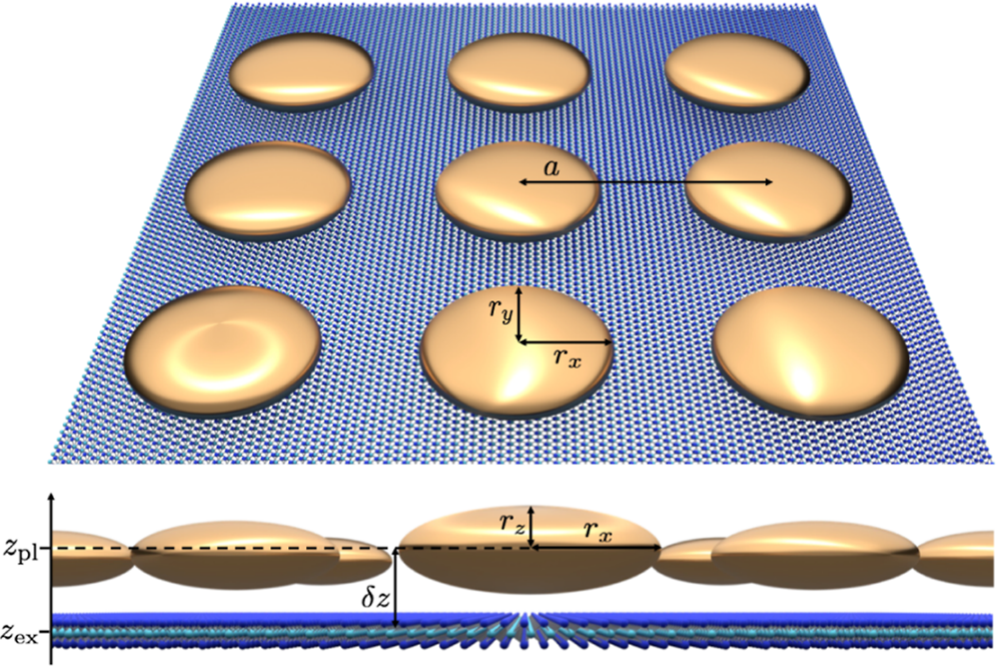
Sketch of the hybrid system: the 2D semiconductor (TMDC) is covered by a square-structured 2D PC of metal nanodisks with the example of gold. The structure is periodic and infinite in the *xy*-plane and embedded in a surrounding medium with constant permittivity ε.

In the context of our study, we adopt a definition of “strong coupling” where the interaction strength between the two systems^36,37^ exceeds the total losses of the system, where the former is quantified by an effective Rabi splitting Ω_eff_ ≈ 2*g*_eff_ > γ_ex_ + γ_pl_^31,32,38^ with *g*_eff_ an effective exciton–plasmon interaction strength and γ_ex/pl_ the individual damping rates. In a classical theory, this is usually termed normal mode splitting, though the underlying dissipative modes are quasinormal modes with complex eigenfrequencies, which are well characterized by Maxwell’s equations.^27^ This definition is particularly relevant for exciton–plasmon coupling as the losses in the plasmonic components are significantly larger than the losses in the excitonic component.^27^ In this work, we study the impact of a two-dimensional (2D) PC on excitonic dynamics in TMDCs. A sketch of the hybrid system is depicted in Figure 1. The semiconductor is located parallel to the *xy*-plane. The 2D PC (square array, lattice constant *a*) of gold disks with semiaxes *r*_*x*_, *r*_*y*_, and *r*_*z*_ is placed on top of the TMDC. The distance between the center of a gold disk and the TMDC is labeled with δ*z* = |*z*_pl_ – *z*_ex_|. The depicted vertical gap between TMDC and PC avoids direct electrical contact which would give rise to hot electron injection^39−42^ but that would also suppress any strong coupling effects.^43^ Experimentally, a similar configuration for relatively large nanoparticles with an in-plane radius of more than 50 nm was already realized in refs (44–47), where TMDCs are coupled to 2D PCs with different lattice structures.

To theoretically study the exciton–plasmon coupling, we develop a semianalytical self-consistent Maxwell–Bloch theory for the hybrid structure in Section 2. We first give a short review on the solution of the Maxwell’s equations (Section 2.1), the description of the TMDC monolayer with the excitonic Bloch equation (Section 2.2) and the 2D PC using Mie theory (Section 2.3). This allows us in Section 2.4 to use the plasmonic dipole density within the PC as a source for the excitonic dipole density of the TMDC, by inserting it into the excitonic Bloch equations. The resulting formulas are Bloch equations that describe the dynamics of excitons in the presence of an electric field and the field-mediated interaction. We highlight the qualitatively different behaviors of momentum-dark excitons that couple to the plasmon-enhanced electric near-field and originally bright excitons with vanishing center-of-mass momentum. Finally, an expression for the emitted electric field is derived from which we can deduce transmission, reflection, and absorption of the hybrid structure for a plane wave excitation.

Using this framework allows us to obtain the absorption spectra of the hybrid system, as shown in Section 3. Our analysis covers the broad range of coupling regimes, varying from weak to strong. In the strong coupling case, we find an additional peak at the unperturbed exciton energy, which exhibits a strong dependence with the temperature and coupling strength. In contrast to conventional Maxwell solvers like ANSYS Lumerical (finite-difference time-domain) or COMSOL (finite elements), the semianalytical theory not only offers substantial reductions in numerical costs but also provides novel physical insights into the exciton–plasmon interaction. Specifically, our semianalytical theory suggests that the additional mode stems from the originally bright excitons that do not participate in the strong coupling to the plasmon-enhanced electric near-field. The semianalytical approach in particular helps to unravel the unperturbed excitonic mode which is weakly broadened in the low temperature limit, i.e., spectrally sharp compared to the normal mode splitting and hard to resolve for pure numerical Maxwell solvers. We find that this excitonic mode is uniformly distributed in real space, thus also located in the immediate vicinity of the MNPs. The observed strong coupling between TMDCs and MNPs or PCs is based on the coupling between plasmon and momentum-dark excitons. We note that there is ongoing discussion regarding exciton–plasmon coupling in nanoshells, with some studies also suggesting the existence of an undisturbed excitonic mode.^48^ To the best of our knowledge, this mode has not been observed in experiments for nanoshells.^49^ However, our results agree with recent experiments^46^ which observe the presence of such an additional excitonic peak for TMDC-PC hybrids.

## 2 Theoretical Model

In this section, we develop a theoretical framework that describes the interaction between TMDC excitons and PC plasmons via the radiation field.

### 2.1 Maxwell’s Equations

Starting from Maxwell’s equations, assuming a nonmagnetic and isotropic medium, the wave equation for the electric field reads
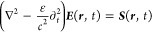
1with the velocity of light *c* and the source
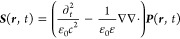
2which is valid for a freestanding sample with dipole density ***P***(***r***,*t*) embedded in a homogeneous, isotropic, and nondispersive dielectric environment with permittivity ε.

A solution of eq 1 can be formally obtained via the scalar Green’s function *G*(***r***, ***r***′, *t* – *t*′)
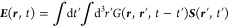
3where the scalar Green’s function depends on the boundary conditions. Both layers of the hybrid structure, cf. Figure 1, are assumed to be aligned parallel to the *xy*-plane with a discrete translation invariance for the PC. Motivated by the symmetry, we apply a Fourier transform only to the in-plane coordinates *x* and *y* and time *t*, keeping all degrees of freedom in our calculation:
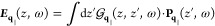
4where we introduced the new Green’s dyadic  that depends on the frequency ω and the in-plane Fourier component **q**_∥_. It converts the dipole density at position *z*′ into an electric field, describing its propagation from *z*′ to the observation position *z*. The term  can be derived analytically^50,51^
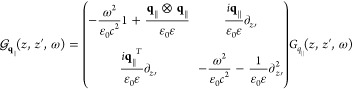
5

Equation 5 gives the Green’s dyadic in Cartesian coordinates regarding the *z*-direction whereas the in-plane components are expressed independent of a special basis for **q**_∥_, which will be specified in Section 2.4. The scalar Green’s function for a constant surrounding permittivity is
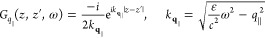
6with wavevector *k*_**q**_∥__ where we abbreviate the absolute value of a vector by, e.g., |**q**_∥_| = *q*_∥_.

Next, using the thin-film approximation for the individual layers,^52,53^ i.e., with TMDC (ex) and PC (pl), then
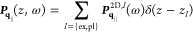
7allows to find an algebraic solution of Maxwell’s equations in Fourier space. Adding the incident field  as a solution of the homogeneous wave equation, eq 1, yields
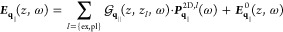
8which allows us to compute the transmission and reflection. Thus, we can connect the dipole density to experimentally measurable far-field signals.

We assume a plane wave excitation that is propagating in the positive *z*-direction
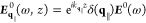
9with an amplitude perpendicular to the *z*-axis ***E***^0^ ⊥ ***e***_*z*_. In the far-field limit, only a *q*_∥_ = 0 Fourier component occurs (see eq 36). The transmission is given by
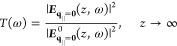
10and the reflection is
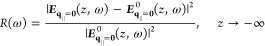
11

The absorption is then easily obtained from
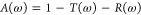
12

### 2.2 Excitonic Dipole Density

To describe the response of the TMDC excitons to the electric field, we define the macroscopic 2D dipole density^52,54^
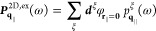
13with the dipole moment ***d***^ξ^, ξ = +/– corresponding to the *K*^+^ and *K*^–^ valley, carrying the circular dichroism.^55^ The strength of the dipole moment ***d***^ξ^ is taken from DFT calculations;^55^ the term  accounts for the value of the 1s excitonic wave function in real space at ***r***_∥_ = **0** and is obtained from the solution of the Wannier equation,^56,57^ incorporating the dielectric environment in the Rytova–Keldysh approximation.^58^ We concentrate on the lowest 1s excitonic state, since it is energetically separated from higher transitions.^5^ A table with all parameters for MoSe_2_ can be found in the Supporting Information. The excitonic transition amplitude is denoted as  and obtained via its Bloch equation in the Fourier domain^59^
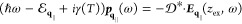
14with the exciton dispersion , the temperature-dependent dephasing rate γ(*T*), the dipole tensor
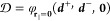
15and the excitonic transition in vector notation
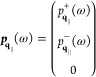
16with center-of-mass wavenumber **q**_∥_ (also referred to as in-plane momentum). The zero in the *z* component is added for convenience to allow for the appearance of square matrices.

A detailed derivation is provided in ref (60), where the rotating wave approximation (RWA) is utilized. This approximation imposes a constraint on the light–matter interaction strength, which must be significantly smaller than the system energies. Therefore, the range of exciton–plasmon coupling strength that can be accurately described in this work is limited to *g*_eff_/*E*^pl^ < 0.1 which is below the ultrastrong coupling regime.^17,61,62^

The temperature-dependent dephasing rate γ(*T*) accounts for nonradiative decay, which typically results from exciton–phonon interactions and is calculated microscopically^57^
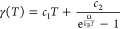
17where *c*_1_, *c*_2_, and the averaged phonon-energy Ω are given in the Supporting Information. All radiative corrections such as lineshift/splitting and broadening are incorporated via the self-consistently calculated electric field ***E***_**q**_∥__(*z*_ex_,ω) given in eq 8.

**Figure 2:**
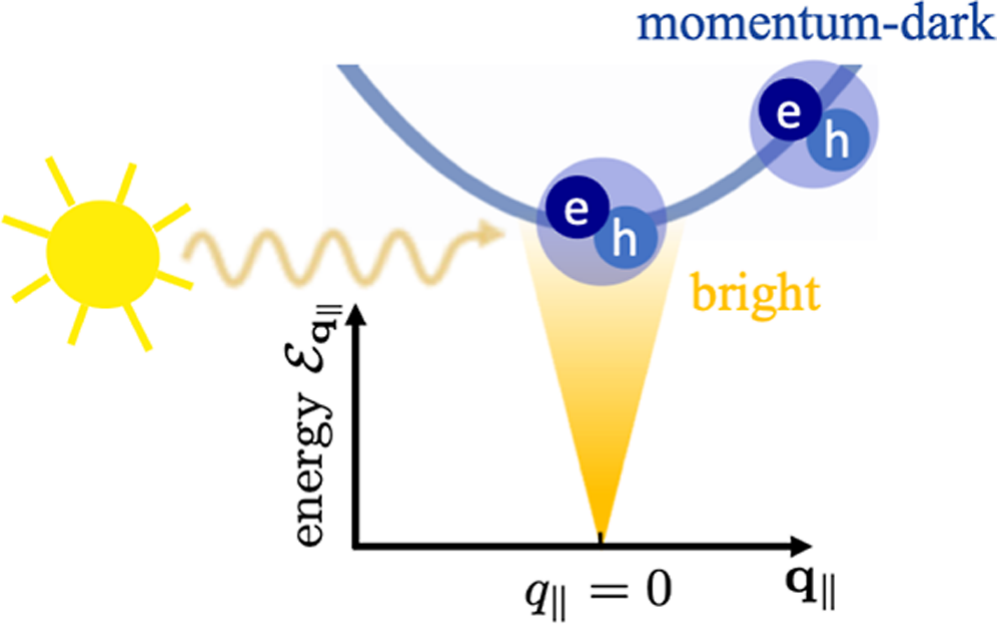
Parabolic exciton dispersion  over center-of-mass momentum **q**_∥_. A bright and a momentum-dark exciton are indicated exemplary. The schematic “sun” represents a radiative excitation from the far-field.

The left-hand side of eq 14 accounts for the dispersion of excitons
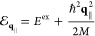
18with, the excitonic mass *M* and the excitonic transition energy *E*^ex^ of 1s-excitons with vanishing momentum. The parabolic exciton dispersion is illustrated in Figure 2. In eq 14, an excitonic transition ***p***_**q**_∥__ can be excited by an electric field ***E***_**q**_∥__ with the same in-plane momentum. The excitonic transition ***p***_**q**_∥__ is called *bright* if it can be excited from the far-field. However, propagating solutions of the wave equation for the electric field, eq 1, are only possible for real values of *k*_**q**_∥__, cf. eq 6, meaning for small momenta *q*_∥_. We refer to excitons with higher momenta as *momentum-dark* as they are inaccessible via far-field illumination.

To connect the solution of eq 14 with the Green’s dyadic formalism solving Maxwell’s equations in the circular basis (+, −), we adjust the notation of eq 13 to
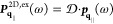
19

Due to the circular dichroism of TMDCs, the dipole tensor is diagonal in a circular polarized basis, .

### 2.3 Plasmonic Polarizability

To describe the response of MNPs, we use Mie–Gans theory,^63−65^ providing a parametric frequency-dependent polarizability **α**(ω). The optical response of every MNP is approximated by a point dipole ***p***^*j*^ at the lattice position ***R***^*j*^
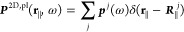
20with
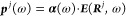
21where ***E***(***R***^*j*^, ω) is the electric field, *excluding* the field generated by the MNP itself which is already incorporated in **α**(ω).

In our system, the dipole approximation for the MNPs is justified by two arguments: First, the MNP gets excited with a plane wave from the far field that couples only negligibly to the higher MNP multipole modes for nanoparticles significantly smaller than the wavelength (*r*_*i*_ ≪ λ).^66−68^ Additionally, the TMDC excitons represent a delocalized 2D continuum of emitters, fostering collective coupling to the MNP where the dipole mode dominates over higher MNP multipole modes, as shown in ref (66). This is further supported by the reduced influence of the quadrupole mode in spheroidal MNPs compared to spheres.^69^ We want to note that, however, for interactions of MNPs with strongly localized emitters such as atoms, molecules, or quantum dots, the dipole approximation might not be sufficient.^66,68,70^

In Cartesian coordinates, with the MNP axes along the corresponding semiaxes (*i* = {*x*, *y*, *z*}), the quasi-static polarizability **α**^qs^ for an oblate spheroid is diagonal with the entries^64,65^
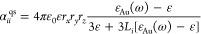
22where *r*_*i*_ is the half axis of the MNP along the corresponding direction, ε_Au_(ω) is the permittivity of gold, ε is the surrounding permittivity, and *L*_*i*_ is the shape factor for oblate spheroids
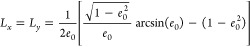
23
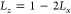
24with the eccentricity
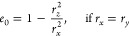
25

To construct the optical response of the MNP-based 2D PC depicted in Figure 1, we need to take the radiative coupling between the nanoparticles into account. Therefore, we consider corrections to the quasi-static Mie–Gans solution of the single particle polarizability in the *modified long-wavelength approximation* (MLWA) including correction terms for dynamic depolarization and radiation damping^71−73^
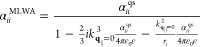
26with the radiative wavevector , compare eq 6. A full self-consistent solution would inherently include radiation damping. However, in eq 21 the electric field generated by the MNP itself is excluded, making this correction necessary. Whether dynamic depolarization and radiation damping are important for a single MNP depends on the size of the MNP: for particle diameters significantly smaller than λ/2π, the static Mie–Gans theory, eq 22, would be a sufficient description. In Section 3, we evaluate the theory for MNPs with in-plane radii of 30 nm, where corrections due to the finite extent become essential for the description of their optical properties.

For the gold-MNP permittivity ε_Au_(ω), as occurring in eq 22, a heuristic, analytical model that fits experimental data for bulk^74^ is parametrized by^75^
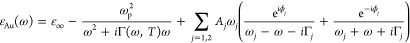
27

The parameters ε_∞_, ω_p_, *A*_*j*_, ω_*j*_, ϕ_*j*_, and Γ_*j*_ are given in the Supporting Information. In the high-frequency limit, the permittivity ε_∞_ of gold differs from unity since *d*-bands in noble metals are filled and provide a high residual polarization.^13^Equation 27 incorporates a Drude-like intraband response of the conduction band and two interband transitions with the possibility to fit asymmetric line shapes in linear bulk spectra (last two terms in eq 27). We combine the room-temperature permittivity given in ref (75) with a temperature and spectrally dependent line width^76−79^ via the damping term Γ(ω, *T*) as a sum of electron–electron and electron–phonon scattering using a Debye model
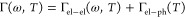
28with^76−79^
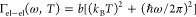
29
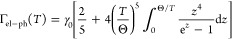
30

A table with all parameters, including the Debye temperature Θ, for the permittivity of gold is given in the Supporting Information. We consider the temperature dependence of the line width only for the Drude contribution in eq 27 since it is the main contribution in the well-studied energy regime below the energy range where interband transitions become relevant, ℏω < 2.4 eV.^79^ For simplicity, we disregard a subtle redshift of ω_p_ for low temperatures. Equation 28 does not consider radiative damping since it is included by self-consistently solving Maxwell’s equations. Equations 21–27 fully describe the response of single nanoparticles to a self-consistently calculated electric field ***E***, eq 8.

In contrast to the single MNP properties, in a PC, the field generated by all other nanoparticles has to be considered self-consistently. The discrete translational invariance in the *xy*-plane allows to collect the interactions with the other MNPs in a modified polarizability  in Fourier space. A self-consistent solution for the two-dimensional dipole density of the 2D PC in coupled dipole approximation is given by ref (80) and experimentally confirmed for gold nanodisks with *r*_*x*_ = *r*_*y*_ ≈ 60 nm and a lattice constant *a* ≈ 500 nm in ref (72). The corresponding dipole density reads
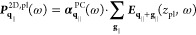
31where the sum includes all reciprocal lattice vectors **g**_∥_ corresponding to Umklapp processes. The effective polarizability tensor  contains corrections due to interactions between the nanoparticles in the coupled dipole approximation restricted to MNP center-to-center distances *a* ≥ 3*r*_*xy*_. In this limit,^81^ the effective polarizability is given by
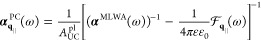
32normalized by the unit cell area *A*_UC_^pl^ of the PC. The form factor
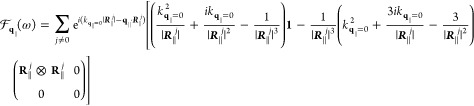
33accounts for particle interactions, with the 3 × 3 unity matrix **1**. The integer *j* indexes the nanoparticles at the in-plane lattice vector positions ***R***_∥_^*j*^ of the 2D PC. Therefore, the form factor depends on the lattice structure and the lattice constant *a*. If the momentum **q**_∥_ equals **0** or a reciprocal lattice vector **g**_∥_, the form factor becomes diagonal with the entries^82^
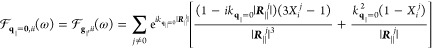
34

The angle dependency of the summands for the entries *i* = *x*, *y*, and *z* is given by *X*_*x*_^*j*^ = cos^2^ θ^*j*^, *X*_*y*_^*j*^ = sin^2^ θ^*j*^, and *X*_*z*_^*j*^ = 0, with the polar angle θ^*j*^ of ***R***_∥_^*j*^. In the numerical evaluation of the form factor, eq 34, the sum is only slowly converging, leading to spurious oscillations. This unphysical result can be circumvented using Ewald’s onefold integral transform,^83^ yielding a fast converging expression for the form factor. An alternative, but numerically more demanding method that describes 2D arrays of particles, including higher multipole orders in full electrodynamic calculations, is given by the extended layer multiple-scattering method.^84−86^ The approach is numerically demanding, particularly when dealing with metallic particles, due to the significant refractive index contrast between the metal and dielectric environment.^86^ In contrast, the method we have employed in our work maintains a good balance between computational efficiency and precision.^72^ Especially, using Ewald’s onefold integral transform^83^ allows us to efficiently calculate the electromagnetic response of 2D PCs and achieves convergence by including less than 100 summands in the calculations.

The optical response of the PC, determined by the effective PC polarizability, eq 32, generally contains two kinds of resonances:^72,82,87^ one is determined by single particle properties, that is the MNP shape, size, and material. A second type of resonance stems from constructive, collective interference between the nanoparticles and can cause very sharp spectral features compared with the broad single particle resonance. In the results Section 3, we choose the MNP and PC parameters to maximize the optical near-field of the PC, such that both resonances appear for the same frequency range. In this case, the collective interactions do not cause a narrow distinct peak, as focused on, e.g., in refs (19, 21, 88–91), but rather amplify and sharpen the present MNP resonance.

Umklapp processes in eq 31 are considered in the near-field of the sample but not in the far-field (|*z* – *z*_pl_| → ∞). This is appropriate since dipole densities with in-plane momentum **q**_∥_ provide purely evanescent electric fields if the wavenumber *k*_**q**∥_ is imaginary, cf. eq 6. It follows that the **g**_∥_ = 0 summand is the only propagating contribution if
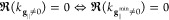
35

The absolute value of the minimal nontrivial reciprocal lattice vector is |**g**_∥_^min^| = 2π/*a* with the lattice constant *a*, as depicted in Figure 1. Consequently, eq 35 can be rewritten as a condition for the lattice constant *a* compared to the wavelength λ of the incoming light
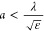
36

If we consider the sum over reciprocal lattice vectors in eq 31 as a diffraction phenomenon, eq 36 states that the lattice constant *a* is small enough that the diffraction pattern in the far-field exhibits only the main maximum and not any additional side lobes. However, for the self-consistent solution, we have to consider all Umklapp processes in eq 31, since both propagating and evanescent electric fields couple to the TMDC excitons, which are located in the near-field of the PC.

### 2.4 Plexcitons

In the following, the electric field, eq 8, and the dipole densities, eqs 13 and 31, are solved self-consistently to provide the near-field and far-field responses of 2D TMDC-PC hybrids to an initially incident plane wave. To solve the set of equations, we insert the dipole densities of the individual layers, eqs 19 and 31, into the solution of the electric field, eq 8, and altogether in the excitonic Bloch equation, eq 14. From this procedure, we obtain a Bloch equation for the excitonic transition ***p***_**q**_∥__
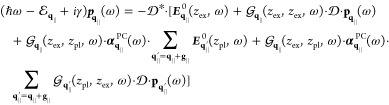
37where the left-hand side describes the free propagation of the excitons, cf. eq 14, and on the right-hand side the self-consistent electric field occurs as a function of the excitonic transition amplitude.

The first term appearing on the right-hand side of 37, after the left parentheses, , accounts for the undisturbed incoming electric field contribution at the TMDC position; the second term carries the renormalization of the exciton line due to radiative self-interactions; the third term accounts for the incoming field  scattered at the PC; and the fourth (final) term is the electric field generated by the excitons and backscattered from the PC. This term is a radiative near-field interaction that couples excitonic transitions with momenta **q**_∥_ to those **q**_∥_^′^ which are shifted by reciprocal lattice vectors **g**_∥_ of the PC compared to the excitonic momentum on the left-hand side. This coupling results from the discretized translational invariance and will later be interpreted as a binding potential for the originally spatially extended excitons. The terms two and four give rise to an intervalley coupling in eq 37, since their prefactor matrices are nondiagonal in the valley index.

Since for the solution of eq 37, we need to consider the observables near the sample, i.e., in a near-field coupling, we apply a quasi-static approximation in eq 5. We neglect propagation effects by setting ω to zero and otherwise only consider a parametric ω-dependency, e.g., in the permittivity ε_Au_(ω), the dipole densities, or the electric field. This approximation is valid as long as^92^
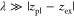
38where the wavelength λ of the incoming light is compared to the distance between the two lattices. Thus, we obtain the simplified (quasi-static) scalar Green’s function
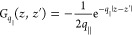
39which shows an exponential decrease that depends on the distance between the source of the electric field at *z*′ and the observer with position *z*. We indicate the quasi-static Green’s function by dropping the dependence on the frequency ω. However, the Green’s dyadic, eq 5, shows that the quasi-static approximation corresponds to neglecting ω^2^ compared to the in-plane momentum *q*_∥_^2^ which is only reasonable if
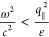
40

In the following, we refer to electric fields and dipole densities that fulfill the condition in eq 40 as *outside the light-cone*. No propagating solution of the wave equation is possible since the electric field is damped exponentially with the distance, cf. eqs 6 and 39. In contrast, in-plane momenta that do not fulfill the condition are *inside the light-cone*. The condition 40 is valid only for excitonic momenta **q**_∥_ belonging to originally momentum-dark excitonic transitions. However, an inspection of eqs 8 and 31 shows that, for a perpendicular plane wave excitation, cf. eq 9, the plasmonic dipole density is only nonzero for in-plane momenta equal to a reciprocal lattice vector **g**_∥_ of the PC. Since only the zeroth order scattering momentum **g**_∥_ = 0 is a propagating solution (inside the light cone), it is allowed to apply the quasi-static approximation for all momenta **q**_∥_ of the excitonic transition, except for **q**_∥_ = **0**. Therefore, we split eq 37 into a near-field Bloch equation (*q*_∥_ > 0) and a radiative contribution (*q*_∥_ = 0) and discuss them separately in the following (Sections 2.4.1 and 2.4.2, respectively). In particular, by inserting the Green’s function for a homogeneous environment permittivity in quasi-static approximation, eq 39, one observes that the right-hand side of eq 37 is proportional to the in-plane momentum. The momentum-dark excitonic transition  (Section 2.4.1) does not couple to the radiative excitonic transition  (Section 2.4.2) and can be solved independently.

#### 2.4.1 Near-Field Exciton–Plasmon Interaction

The resulting Bloch equations for *q*_∥_ ≠ 0 are identical to eq 37, with the quasi-static approximation applied to every Green’s dyadic. To account for the circular dichroism of the TMDC excitons, we chose a circular polarized basis (**e**^+^, **e**^–^, and **e**^*z*^). Any quantity in Cartesian coordinates is transformed to the circular polarized basis by multiplying with the unitary (change-of-basis) matrix 
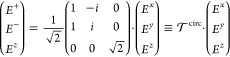
41

In the quasi-static limit, a diagonalization of eq 37 in circular polarized basis corresponding to the valleys *K*^+^ and *K*^–^ is given by a transformation , eq 42, that depends on the polar angle ϕ of the in-plane momentum **q**_∥,_
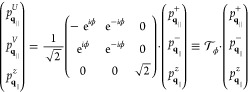
42

The transformation  corresponds to an in-plane rotation of the momentum space, orienting the momentum **q**_∥_ along the *V*-axis and perpendicular to the *U*-axis. The reason for naming the components *V* (conic) and *U* (parabolic) relates to the resulting exciton dispersion and is clarified in eqs 44–46. We find that the transformation into the (**e**^*U*^, **e**^*V*^, and **e**^*z*^) basis is the diagonalization of the in-plane contribution of the quasi-static Green’s dyadic
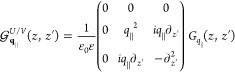
43

Applying the rotation to the Bloch eq 37 finally allows a diagonalization process
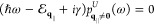
44
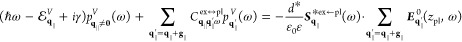
45where eq 44 possesses the undisturbed, parabolic (*U*-shaped) exciton dispersion for . In contrast, we find
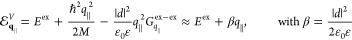
46yielding a conic (*V*-shaped) dispersion for  similar to results reported in ref (93). The excitonic self-interaction term, *q*_∥_^2^*G*_*q*_∥__^ex–ex^, dominates the kinetic energy contribution for a small *q*_∥_. The optical sources of , cf. eq 45, occur on the right. Obviously, the near-field interaction of exciton and plasmon is fully encoded in . The interaction with the PC is denoted by the contributions (ex ↔ pl) and provides a momentum and frequency dependent PC-induced excitonic self-interaction , namely, a plasmon-mediated effective exciton–exciton interaction. For a concise notation without loss of information, we indicate the *z*-dependencies of the prefactor Green’s functions in their superscripts. On the right-hand side of eq 45, the existence of an incoming field , that was scattered at the PC, results in the excitation of the conic excitonic transition. The interaction with the PC provides a coupling to in-plane momenta shifted by reciprocal lattice vector **g**_∥_ of the PC due to Umklapp processes depending on the PC lattice structure. The PC-induced excitonic self-interaction reads
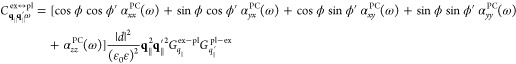
47and the source term that arises from the scattering of the incoming field at the PC becomes
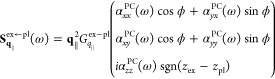
48if we express  in Cartesian coordinates. To achieve these expressions, derivatives of the Green’s function with respect to *z*′ have been carried out, cf. eq 5.

Since we were able to decouple the valleys of the excitonic transition  in eq 37 into a conic (optically driven , eq 45) and an undisturbed parabolic (, eq 44) contribution, we can now solve them independently. The parabolic Bloch equation for  immediately yields
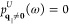
49

In contrast, in eq 45, the conic Bloch equation for  contains a coupling of excitons with different center-of-mass momenta, mediated by the PC. We project the excitonic transition  onto a set of eigenstates  of the conic dispersion with suitable expansion coefficients *p*^λ^
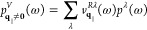
50

The corresponding symmetric, non-Hermitian eigenvalue problem reads similar to ref (26)
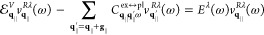
51

As a consequence of the non-Hermiticity we have to distinguish between left and right eigenstates . To justify the projection onto these states, we verify the existence of solutions of eq 51 and their completeness numerically. By applying a suitable normalization, we ensure orthonormality using
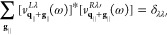
52

With this expression, we project the conic excitonic transition in the Bloch eq 45 on the new states according to eq 50. Substituting the momentum dependencies with the corresponding eigenvalue in eq 51 and taking advantage of the orthonormality condition between the eigenstates finally allows us to give a solution for the conic contribution of the excitonic transition
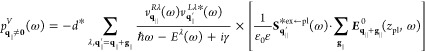
53

To connect the excitonic transition with observables, we insert it in the dipole density of the TMDC layer. The contribution arising from the conic part becomes
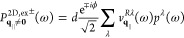
54

The full dipole density stemming from momentum-dark excitons trapped in the plasmonic potential is finally given by
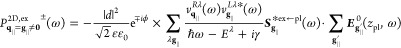
55

For momenta that do not correspond to the wavenumber of the incoming field shifted by a reciprocal lattice vector, the TMDC polarization vanishes due to the absence of a suitable excitation.

#### 2.4.2 Radiative Exciton–Plasmon Interaction

In the previous section, the excitonic dipole density outside the light-cone was derived. However, to determine the far-field response, we need an expression for the dipole density within the light cone, which is a source for propagating solutions of the electric field. Therefore, we evaluate the Bloch eq 37 for *q*_∥_ = 0, i.e., the radiative Bloch equations
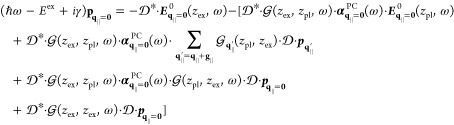
56

For clarity, we suppress the index of the vanishing in-plane momentum **q**_∥_ = **0** in the notation for the radiative Green’s dyadic. The previous example provides us with all the necessary tools to solve this equation. Simplifying the individual terms on the right-hand side is demanding but straightforward by explicitly evaluating the matrix products between the dipole elements, eq 15, the Green’s dyadic, eq 5 and the PC polarizability . Finally, we collect all summands containing  on the left-hand side and multiply with the inverse of its prefactor. This procedure yields
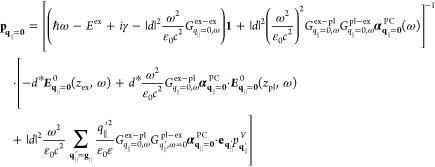
57with the unity vector in polar coordinates
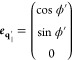
when we express the excitonic transition  and the PC polarizability  in a linear polarized basis.

The denominator of eq 57 corresponds to harmonic oscillators at frequency ω with renormalized eigenenergies
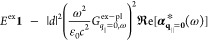
58and the damping
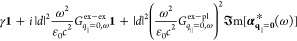


The ex–ex term accounts for the radiative dephasing of the TMDC excitons, whereas the last term governs radiative interference phenomena between the TMDC and the PC. For a resonant TMDC–PC interaction (*E*^ex^ = *E*^pl^), the real part of the PC polarizability can be neglected . This yields an unchanged excitonic eigenenergy, and the coupling to the PC mainly modifies the damping. The numerator includes, from left to right: the direct excitation of excitonic transitions via the incident electric field, the plasmon mediated excitation from the incoming field that was first scattered at the PC, and finally the plasmon-mediated influence of momentum-dark excitonic transitions. From the derived excitonic transition, we deduce the dipole density via eq 19.

#### 2.4.3 Scattered Electric Field

To give explicit results for the electric field emitted by the TMDC layer , we multiply the dipole density, that was derived in the previous section, with the Green’s dyadic. We find the momentum-dark dipole density to be an eigenstate of the quasi-static Green’s dyadic, cf. eq 5. Therefore, we find
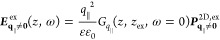
59
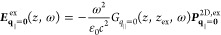
60

In fact, also a *z*-component of the electric near-field  occurs. However, it only causes a nonvanishing *z*-component of the PC diple density since  is assumed to be diagonal regarding its *z*-entries. This *z*-polarization of the PC is not transferred to the far-field for **q**_∥_ = **0**. To obtain the contribution of the PC to the far-field, , we deduce the dipole density of the 2D PC, according to eq 31. It is
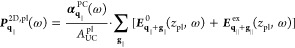
61

As already justified above for dense PCs, cf. eq 36, there is no propagating solution of Maxwell’s equations with an in-plane wavenumber equal to a nontrivial reciprocal lattice vector. Therefore, we may drop the sum over **g**_∥_ ≠ 0 for the exciting field. As for the radiative TMDC contribution, the dyadic Green’s function has to contain the full time dependency. Furthermore, since *z* ≠ *z*′, the *z*-entries of the Green’s dyadic vanish. The in-plane far-field plasmon contribution reads
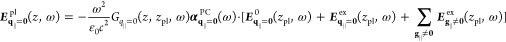
62

Adding all contributions of the electric field according to eq 8, results in
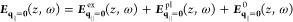
63

#### 2.4.4 Overview of the Theory

**Figure 3:**
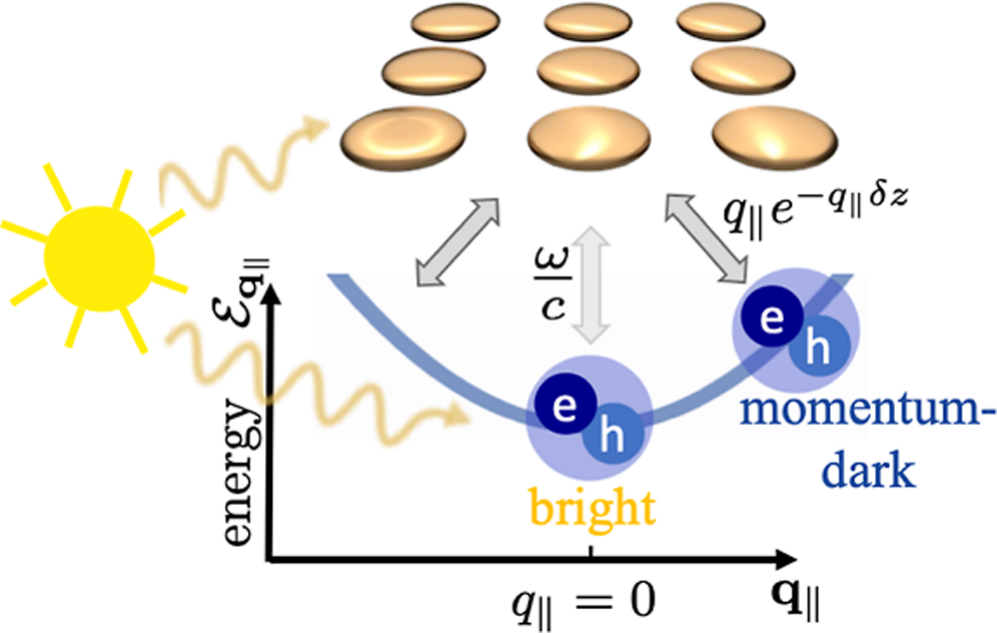
Schematic of the theory: the PC couples to the bright and momentum-dark TMDC excitons which are illustrated on the parabolic dispersion curve . The sun represents the far-field excitation. The gray arrows account for the exciton–plasmon coupling, with the indicated coupling strengths.

With the theoretical framework now fully established, our attention turns to shedding some light on the physical meaning of the derived equations. Figure 3 provides a schematic of the theory. We excite the TMDC-PC hybrid via far-field illumination , eq 9, represented by the sun-like schematic in Figure 3. It directly acts on the PC plasmons, eq 61, and the bright excitons , eq 57. The scattering Umklapp processes of the incoming light at the PC provide access to momentum-dark excitonic transitions, eq 45, via the plasmon-enhanced electric near-field. These interactions are typical dipole–dipole near-field interactions, proportional to , which can be seen by explicitly inserting the quasi-static Green’s function, eq 39, into the conic Bloch equation, eq 45. In this expression, δ*z* = |*z*_pl_ – *z*_ex_| constitutes the vertical center-to-center distance of TMDC and MNPs, pointing out the importance of a small gap-size for maximizing the coupling strength as well as the choice of metal nanodisks with a small out-of-plane half axis *r*_*z*_ as depicted in Figure 1. The coupling between the plasmon and the bright exciton behaves qualitatively differently with the coupling strength significantly reduced, proportional to ω/*c*, cf. eq 57. The implications of these different coupling mechanisms are numerically evaluated in Section 3. In the spirit of ref (94), we classify our system after the diagonalization of the two exciton modes as a semiclassical description of two strongly coupled oscillators (plasmon and (*q*_∥_ > 0)-excitons) and one additional weakly coupled oscillator (*q*_∥_ = 0)-exciton. Finally, we note that, in the limit *a* → ∞, the developed theory is also applicable for a TMDC coupled to a single MNP without qualitatively changing the results.

## 3 Numerical Results and Discussion

We numerically evaluate the key eqs 62, 60, 59, 55 and 51 to calculate the absorption, eq 12, of the hybrid structure. If not stated differently, we perform calculations for a MoSe_2_–PC stack with the geometry specified in Table 1. The given parameters yield a PC with its resonance energy at *E*^pl^ = 2.03 eV. We choose the lattice constant *a* in a way that the collective PC resonance sharpens the single MNP response that is governed by the bulk metal parameters and aspect ratio *r*_*z*_/*r*_*x*_. Material specific parameters for gold and MoSe_2_ are given in the Supporting Information.

**Table 1:** Geometry of TMDC-PC Hybrid

*r*_*x*_	30 nm	*a*	10 *r*_*x*_
*r*_*y*_	30 nm	δ*z*	11 nm
*r*_*z*_	10 nm	ε	2.4

### 3.1 Coupling Regimes at Room Temperature

**Figure 4:**
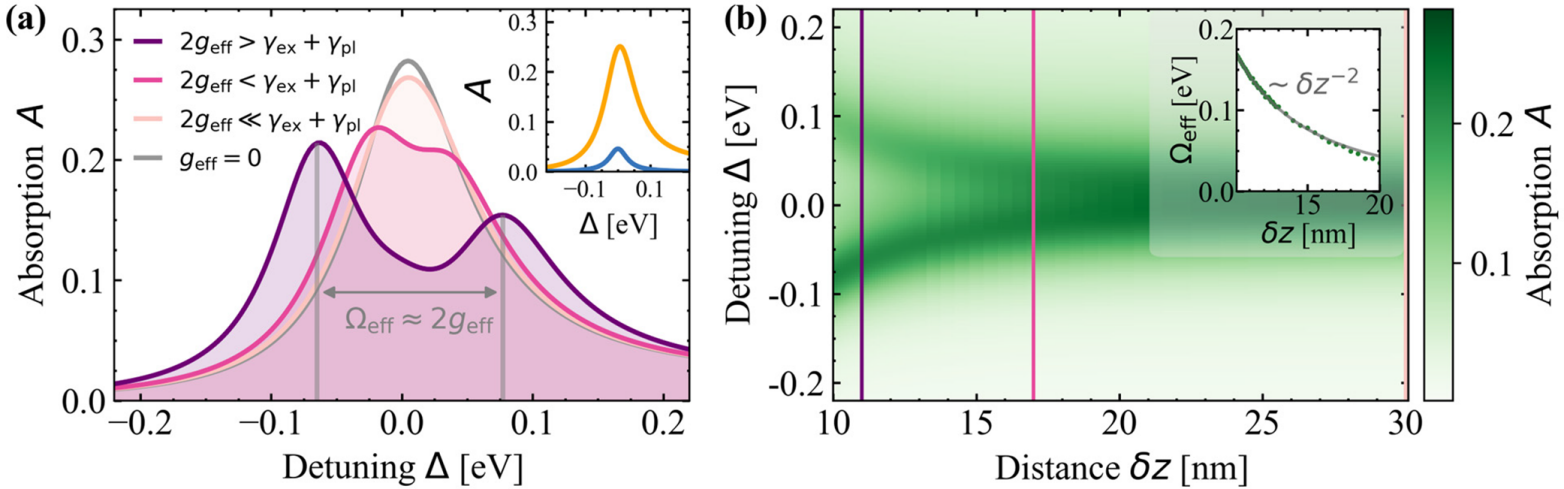
Absorption for different center-to-center distances δ*z* = |*z*_pl_ – *z*_ex_| between TMDC and PC at room temperature, corresponding to a scan over an effective coupling strength *g*_eff_. (a) Absorption spectra for δ*z* = [11, 17, 30]nm belonging to different interaction regimes, from weak (2*g*_eff_ < γ_ex_ + γ_pl_) to strong (2*g*_eff_ > γ_ex_ + γ_pl_) coupling. For comparison, the artificially uncoupled case (gray), is the sum of single TMDC (blue) and PC (yellow) absorption in the inset. (b) Color map of the absorption depending on the distance δ*z* and the detuning Δ. The vertical lines indicate the spectra shown in panel (a). Inset: effective Rabi energy Ω_eff_ over δ*z*. It reaches values significantly larger than 100 meV. The peak splitting extracted from the full theory is compared to a fit *b*/(δ*z*)^2^ with *b* = 17.24 nm^2^eV.

To study different coupling regimes, the modification of the layer distance δ*z* allows direct access to the strength of the near-field-mediated interaction. Figure 4 shows the absorption of the hybrid over the detuning Δ = ℏω – *E*^pl^ between the electric field and the plasmon for the resonant case of exciton and plasmon (*E*^ex^ = *E*^pl^) for different exciton–plasmon distances δ*z*. Modifying the distance δ*z* qualitatively changes the hybrid’s spectrum. All cases shown in Figure 4 have been observed in experiments with TMDC excitons coupled to MNPs or PCs, yet lacking a microscopic theory, which we provide from an excitonic perspective. It is important to note that with respect to the classification of coupling regimes (weak, strong, or ultrastrong coupling),^94^ we classify our approach to be valid in the semiclassical weak and strong coupling limits.

#### 3.1.1 Weak Coupling (δ*z* = 30 nm, δ*z* = 17 nm)

For MNP-TMDC distances where δ*z* is approximately equal to or exceeds the effective extension of the MNP, the exciton–plasmon coupling is weak. Thus, for δ*z* ≥ 30 nm, the line shape is qualitatively preserved compared to the uncoupled case. However, the interaction induces an additional damping, increasing the line width and reducing the maximal absorption. This is because the interaction terms in the denominators of eqs 55 and 57 are imaginary, thus effectively acting like an additional damping to the phonon damping γ. This behavior is analogous to the classical coupled oscillator model^94,95^ and has been experimentally observed between TMDC excitons and MNP plasmons in refs (29 and 43).

With further decreasing of the TMDC-PC distance, the absorption line shape changes. The absorption of the hybrid develops two maxima, while the absorption at the original, nonperturbed, resonance energy shrinks. The splitting between the formed maxima can be identified with an effective Rabi energy Ω_eff_. Its distance dependency is depicted in the inset of Figure 4b, illustrating the (δ*z*)^−2^ proportionality. To assign the hybrids to a particular coupling regime, the effective Rabi energy Ω_eff_ ≠ 0 is compared with the line width γ_ex_ and γ_pl_ of the individual constituents.^94,96^ A coupling strength leading to a peak splitting that is small compared to the line widths as, e.g., in the δ*z* = 17 nm^89,90^ case is still assigned to weak coupling and has been observed in experiments with TMDCs coupled to a MNP or a PC in refs (28, 44, 46, and 97).

#### 3.1.2 Strong Coupling (δ*z* = 11 nm)

Further decreasing the distance of TMDC and PC allows one to enter the strong coupling regime as Ω_eff_ > γ_pl_ + γ_ex_ with peak splittings up to Ω_eff_ ≈ 140 meV. In this case, mathematically, in eqs 51–55, a real part is added to the excitonic eigenenergy, thus changing the root of the denominator in eq 55, and consequently the plexcitonic resonance energies. Experimentally, strong coupling has been observed up to room temperature^29−32,43,47,98−100^ for TMDC-MNP hybrids with effective Rabi energies similar to the values shown in Figure 4.

It would be worth also considering the possibility of ultrastrong coupling in a similar platform. However, this limit cannot be addressed by the developed theory due to the constraint on the coupling constant *g*_eff_/*E*^pl^ < 0.1, since the RWA is applied (Section 2).

### 3.2 Low Temperatures

**Figure 5:**
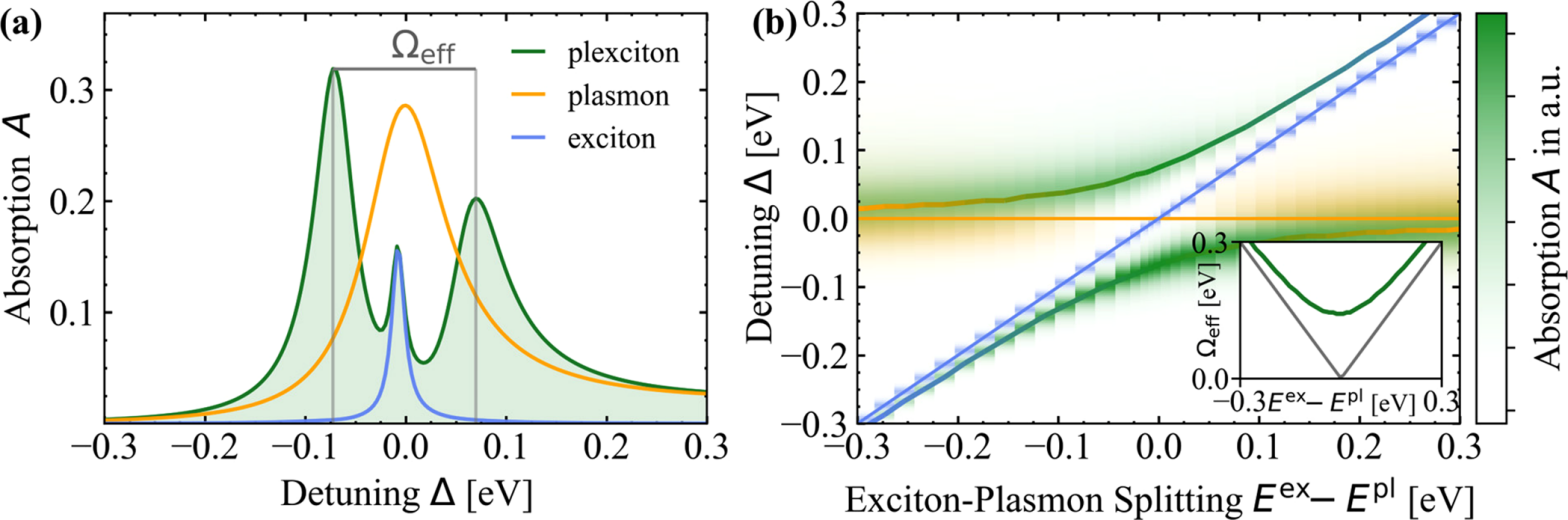
(a) Absorption spectrum at liquid nitrogen temperature *T* = 77 K of the hybrid structure (green) compared to the uncoupled absorption of the TMDC (blue) and the 2D PC (yellow) for *E*^ex^ = *E*^pl^. The effective Rabi energy Ω_eff_ ≈ 140 meV is identified as the energetic separation between the two outer plexcitonic resonances. (b) Dispersion of the plexciton branches depending on the excitonic resonance energy *E*^ex^. The upper and lower branches are highlighted with solid lines as a guide to the eye. The hybrid spectrum shows an exciton–plasmon splitting, where plasmon (yellow) and exciton (blue) hybridize to plexcitonic modes (green). The horizontal and main diagonal mark the undisturbed plasmon (yellow), respectively, exciton (blue) resonances. Inset: splitting Ω_eff_ (green) between plexcitonic branches depending on the difference between the resonance energies *E*^ex^ – *E*^pl^. The gray linear plot indicates the splitting without any interaction.

To study low-temperature effects, Figure 5a depicts the absorption spectrum in the strong coupling case for coinciding exciton and plasmon resonances *E*^ex^ = *E*^pl^ at liquid nitrogen temperature *T* = 77 K. In addition to the typical strong coupling spectrum, *we find a third peak at the undisturbed exciton resonance*. At room temperature (Figure 4a) this peak is not well resolved due to the increased damping. To investigate the dispersion of the three-peak spectrum, we numerically vary the exciton energy around the plasmon resonance in Figure 5b. The color coding breaks the full absorption of the hybrid down to exciton (blue), plasmon (yellow), and the hybridized plexciton contribution (green). The individual plasmon and exciton dispersions are indicated by the horizontal line and the main diagonal, respectively. The avoided-crossing behavior of the upper and lower branches substantiates that the system is indeed in the strong coupling regime. However, the spectral position of the additional middle peak coincides with the unperturbed exciton energy *E*^ex^. The inset depicts the energy separation of the hybrid branches, where the minimum is the effective Rabi energy of the exciton and plasmon. For comparison, the linear gray plot indicates the energy separation between the exciton and plasmon without any interaction.

#### 3.2.1 Interpretation of the Bright Excitonic Mode

The additional peak in Figure 5a,b stems from the bright excitonic transition ***p***_**q**_∥__ with in-plane momenta **q**_∥_ inside the light cone, see eq 40 and Figure 3. It is caused by the qualitative difference between the exciton–plasmon coupling strength for bright and momentum-dark excitons that was already discussed in Section 2.4.4. With our restriction to PCs that fulfill eq 36, the only nonvanishing contribution to the third peak is , eq 57, meaning it constitutes a weakly coupled bright excitonic mode.

The appearance of the bright excitonic mode in Figure 5a can be traced back to a qualitative difference of our description to previous models.^27,33,95,101^ It results from the geometry of the considered subsystems: the PC consists of discrete dipoles that feature scattering processes. This allows the plasmon to couple to excitonic transitions outside the light cone via a plasmon-enhanced electric near-field. In contrast, the TMDC facilitates continuous translational invariance. Therefore, it is necessary to distinguish between excitonic transitions with in-plane momenta in- and outside the light cone, providing that  does not couple to the plasmon-enhanced near-field but only to its **q**_∥_ = **0** Fourier mode.

As plasmons are popular for amplification of the electric field in their vicinity, the near-field exciton–plasmon interaction is much stronger than the radiative (far-field) contribution. However, the **q**_∥_ = **0** Fourier component is the propagating mode, also transferring the plasmon response to the far-field. It is physically intuitive, due to the argument of energy conservation, that this mode cannot provide a field enhancement and thus yields a reduced coupling strength to the TMDC excitons.

In Figure 5a, we find a subtle damping and broadening due to the radiative exciton–plasmon interaction for the excitonic mode compared to the free-standing excitonic TMDC absorption (blue), similar to the weak exciton–plasmon coupling regime.

We want to note that trions may cause similar looking spectrally sharp features at low temperatures as observed in refs (99, 100, and 102) that, however, originate from fundamentally different physics.

#### 3.2.2 Visibility of the Bright Excitonic Mode

**Figure 6:**
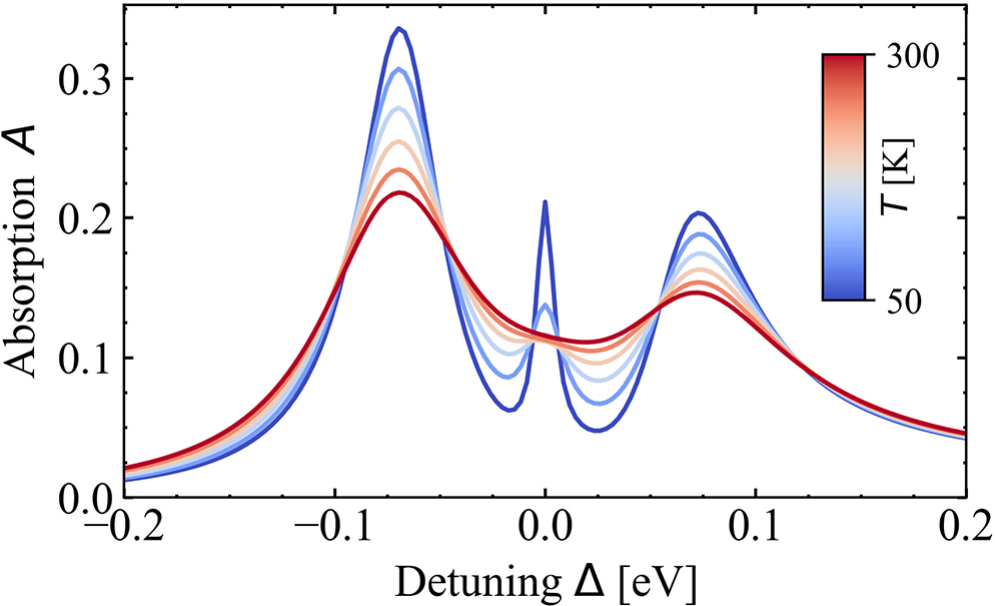
Absorption for different temperatures *T* between 50 and 300 K in the case of coinciding exciton and plasmon resonance. For room temperature *T* = 300 K (red), the hybrid features a typical strong coupling spectrum.

Comparing Figures 4 and 5, we find that the visibility of the weakly coupled excitonic mode strongly depends on the temperature. This trend is analyzed in Figure 6. It illustrates the hybrid absorption spectrum for several temperatures between *T* = 50 K and *T* = 300 K with the excitonic mode clearly visible for low temperatures. With increasing temperature, the absorption peak shrinks until the increasing exciton damping reaches values of approximately half of the peak splitting at room temperature. The larger the peak splitting Ω_eff_, that in turn characterizes the coupling strength, the smaller is the plexcitonic absorption contribution at Δ = 0, cf. Figure 4, which improves the visibility of the excitonic mode.

The bright excitonic mode, beyond the usually found peak splitting,^28−33,44,97,103^ was recently observed in ref (46) at cryogenic temperatures. Measured was the differential reflection of a WSe_2_-monolayer, encapsulated in hBN, on top of a PC with lattice constant *a* = 300 nm consisting of gold nanodisks with radii *r*_*x*_ = *r*_*y*_ ≈ 60 nm, *r*_*z*_ ≈ 8 nm and TMDC-PC distance δ*z* = 12 nm. To the best of our knowledge, this was the first observation of the additional excitonic mode since most experimental works operate either at room temperature or with the interaction strength too small.

#### 3.2.3 Comparison to Previous Models

Previous theoretical analysis was restricted to a phenomenological coupled oscillator model^95^ and the Jaynes-Cummings model^94^ applied on TMDC-MNP and PC hybrids^28−30,32,43−47,99,100,103^ or to near-field excitation involving quasinormal modes.^27,101,104^ Numerical approaches using Maxwell solvers have been used to confirm theoretical and experimental results at room temperature.^27−32,45−47,105,106^ The theory developed in this contribution provides a solution for the electric near- and far-fields explicitly and exceeds the limits of phenomenological models by giving a microscopic description of the TMDC excitons, including the exciton center-of-mass momentum to derive the coupling strength.

When included in the coupled oscillator model, the additional excitonic mode was described as a third oscillator and assigned to excitons spatially separated from the electric field hot spots caused by a single MNP^30^ or a PC.^46^ In the case of molecules that feature Frenkel excitons, coupled to plasmons, this is a proper explanation. Frenkel excitons are localized at the respective molecule, which means that they cannot strongly couple to plasmons unless the molecule is located within an electric field hotspot of the plasmonic structure. For Frenkel excitons, this effect is well-known and frequently observed, as, e.g., in refs (107–111). To a certain extent, this phenomenological explanation is also consistent with the developed microscopic theory for Wannier excitons since the strong coupling is restricted to **q**_∥_ ≠ **0** Fourier modes, corresponding to spatially localized states.^26^ However, the **q**_∥_ = 0 mode, responsible for the bright excitonic mode, constitutes a constant spatial distribution in real space. Our analysis therefore shows that the additional excitonic mode  is uniformly distributed in the TMDC plane, also in the vicinity of the MNPs.

Another interesting feature of the absorption spectra is the asymmetry of the spectral peak line shape with respect to width and height. The asymmetry is also covered by phenomenological models^94,95^ and is consistently observed in experiments.^28−31,43,45,100^ One reason for the asymmetry are different line shapes, line widths, and dipole moments of exciton and plasmon. However, within our microscopic model, another factor contributes to the observed asymmetry. The momentum-dark excitons responsible for the peak-splitting exhibit energy distributions up to 50 meV on the exciton dispersion, eq 18, well above the bright exciton state. Consequently, these excitons, which facilitate the strong exciton–plasmon coupling, are detuned from the joint plasmon and bright exciton resonance. The magnitude of this energy difference depends on the dielectric environment, cp. eq 18, and the distance δ*z*, as discussed in Section 2.4.4. The detuning leads to an incomplete hybridization between the plasmons and excitons. Consequently, the peak at lower energies effectively contains more plasmonic contributions, which results in a stronger absorption, whereas the peak at higher energies is shaped by the momentum-dark excitons. This leads to a broadening on its high-energy side, where the energy distribution on the excitonic dispersion influences the absorption profile.

## 4 Conclusions

We have introduced a self-consistent theory that highlights the extraordinary optical properties that can be achieved by combining ultrathin semiconductors, such as transition metal dichalcogenides (TMDCs), with plasmonic crystals (PCs) composed of metal nanoparticles (MNPs).

We developed a theoretical formalism to describe the coupling between a TMDC and a 2D PC, mediated by the self-consistently solved electric field. More explicitly, we considered the optical response of collective plasmons of 2D PCs within the excitonic Bloch equation. The arising effective intra- and intervalley exciton–exciton interactions for momentum-dark excitons have been decoupled in the eigenbasis of the quasi-static Green’s dyadic into excitonic branches with conic and parabolic dispersion. Any near-field effects appeared to be incorporated via the conic contribution. In contrast, momentum-bright excitons show qualitatively different behaviors, as the coupling strength is proportional to their negligible center-of-mass momenta. Nevertheless, signatures of the momentum-dark excitons are visible in the far-field signal due to a second scattering process at the PC. By evaluating the absorption for different TMDC-MNP separations, we were able to compare different coupling strengths and observe weak and strong coupling line shapes, the latter obeying an avoided-crossing dispersion behavior. We also find new features in the hybrid spectrum beyond the phenomenological model of two coupled oscillators:

The momentum dependency of the exciton dispersion and the coupling strength are responsible for the characteristic asymmetric line shape of TMDC-MNP hybrid spectra. Furthermore, the momentum-bright TMDC excitons do not participate in the strong coupling due to their reduced coupling strength but emit undisturbed into the far-field, resulting in a third peak in the hybrid spectrum at low temperatures.

Thus, our approach yields new analytical insights into exciton–plasmon coupling and allows to significantly reduce the computational costs compared to numerical Maxwell solvers. Numerical approaches, while providing a more accurate description of the electromagnetic field, do not allow for the analytical derivation of effective coupling constants between excitons and plasmons and eventually result in spectrally sharp features, which is a key aspect of our work.

The theory developed in this paper lays a strong foundation for future research: hybrids incorporating plasmonic structures are, due to field confinement, particularly intriguing for the excitation of excitonic nonlinear optical effects, allowing for lower incident electric field strength and effective wave mixing without the necessity of phase matching.^112^ This has potential applications in harmonic generation, nonlinear propagation, and all-optical switching.^113^ A notable application in hybrids of TMDCs and metal nanostructures is enhancing TMDC higher harmonic generation, which was experimentally already realized in ref (114). Our framework for describing the electric field could encompass nonlinear optical effects; however, this would require considering nonlinearities in the equations of motion for both excitons,^60^ leading to bleaching, energy renormalizations, and higher-order Coulomb correlations,^60^ and plasmons. Another promising future research direction would be a semianalytical derivation of photoluminescence, tailored for TMDC–plasmon interactions. Such a study would necessitate to combine the detailed excitonic framework^115,116^ with a quantized treatment of the electric field in the plasmonic component, e.g., quasi-normal mode quantization.^101,104,117−119^ This is particularly exciting as the exciton–plasmon interaction allows for Purcell enhancement of the exciton photoluminescence, a phenomenon with critical implications for applications such as single photon emission.^120^

## Supporting Information Available

Materials and model parameters (PDF)
