# Supplementary material for: Strong Coupling of Two-Dimensional Excitons and Plasmonic Photonic Crystals: Microscopic Theory Reveals Triplet Spectra
Source: ACS Photonics. 2024 Mar 27;11(4):1396–411. doi: 10.1021/acsphotonics.3c01208 (PMC11027155; doi:10.1021/acsphotonics.3c01208)
Supplement: Supplementary file 1 — ph3c01208_si_001.pdf [file ph3c01208_si_001.pdf]

Supporting Information for Strong Coupling of Two-Dimensional Excitons and Plasmonic Photonic Crystals: Microscopic Theory Reveals Triplet Spectra

Lara Greten,[*,†] Robert Salzwedel,[†] Tobias Göde,[†] David Greten,[‡] Stephanie Reich,[¶] Stephen Hughes,[§] Malte Selig,[†] and Andreas Knorr[*,†]

†*Nichtlineare Optik und Quantenelektronik, Institut für Theoretische Physik, Technische Universität Berlin, 10623 Berlin, Germany*

‡*Formerly: Institut für Theoretische Physik, Technische Universität Berlin, 10623 Berlin, Germany*

Current address: Fritz Haber Institute of the Max Planck Society, Theory Department, 14195 Berlin, Germany

¶*Experimentelle Festkörperphysik, Freie Universität Berlin, 14195 Berlin, Germany*

§*Department of Physics, Engineering Physics and Astronomy, Queen's University, Kingston, Ontario K7L 3N6, Canada*

E-mail: lara.greten@tu-berlin.de; andreas.knorr@tu-berlin.de

A Material and Model Parameters

The parameters for numerical computations are mainly given in semiconductor units to improve the numerical accuracy which use fs and nm as a measure of time and length, eV for energy and the elementary charge e.

The excitonic wavefunction $\varphi_{\mathbf{r}_\parallel=\mathbf{0}}$ appears as the solution of the Wannier equation, similar to Refs. 3,4 for the chosen dielectric environment.

Table S1: universal constants in semiconductor units

c	299.7925 nm/fs
$\hbar$	0.658212196 eV fs
k_B	0.0861745 meV/K

Table S2: Parameters for $MoSe_2$

d	$\varphi_{\mathbf{r}_\parallel=\mathbf{0}} \cdot 0.25\, e\,\mathrm{nm}$ [1]
$\varphi_{\mathbf{r}_\parallel=\mathbf{0}}$	$0.46\,\mathrm{nm}^{-1}$
M	$6.1\,\mathrm{eV\,fs^2 nm^{-2}}$ [2]
c_1	0.091 meV/K [3]
c_2	15.6 meV [3]
Ω	30 meV [3]

Table S3: Parameters for the permittivity of gold taken from Ref. 5 (converted to semiconductor units)

ϵ_∞	1.53
ω_p	$12.99\,\mathrm{fs}^{-1}$
ω_1	$4.02\,\mathrm{fs}^{-1}$
ω_2	$5.69\,\mathrm{fs}^{-1}$
Γ_1	$0.82\,\mathrm{fs}^{-1}$
Γ_2	$2.00\,\mathrm{fs}^{-1}$
A_1	0.94
A_2	1.36
ϕ_1	$-\pi/4$
ϕ_2	$-\pi/4$

Table S4: Parameters for temperature dependent linewidth of gold taken from Ref. 6 (converted to semiconductor units)

b	$0.6329\,\mathrm{eV}^{-1}$
γ_0	$0.0219\,\mathrm{eV}$
Θ	$185\,\mathrm{K}$

References

(1) Xiao, D.; Liu, G.-B.; Feng, W.; Xu, X.; Yao, W. Coupled Spin and Valley Physics in Monolayers of MoS_2 and Other Group-VI Dichalcogenides. *Physical Review Letters* **2012**, *108*, 196802.

(2) Kormányos, A.; Burkard, G.; Gmitra, M.; Fabian, J.; Zólyomi, V.; Drummond, N. D.; Fal'ko, V. $\mathbf{k} \cdot \mathbf{p}$ theory for two-dimensional transition metal dichalcogenide semiconductors. *2D Materials* **2015**, *2*, 022001.

(3) Selig, M.; Berghäuser, G.; Raja, A.; Nagler, P.; Schüller, C.; Heinz, T. F.; Korn, T.; Chernikov, A.; Malic, E.; Knorr, A. Excitonic linewidth and coherence lifetime in monolayer transition metal dichalcogenides. *Nature Communications* **2016**, *7*, 13279.

(4) Berghäuser, G.; Malic, E. Analytical approach to excitonic properties of MoS_2. *Physical Review B* **2014**, *89*, 125309.

(5) Etchegoin, P. G.; Le Ru, E. C.; Meyer, M. An analytic model for the optical properties of gold. *The Journal of Chemical Physics* **2006**, *125*, 164705.

(6) Liu, M.; Pelton, M.; Guyot-Sionnest, P. Reduced damping of surface plasmons at low temperatures. *Physical Review B* **2009**, *79*, 035418.
